# HAS2-AS1 is a novel LH/hCG target gene regulating HAS2 expression and enhancing cumulus cells migration

**DOI:** 10.1186/s13048-019-0495-3

**Published:** 2019-02-28

**Authors:** Yuval Yung, Libby Ophir, Gil M. Yerushalmi, Micha Baum, Ariel Hourvitz, Ettie Maman

**Affiliations:** IVF Unit and Reproduction Laboratory, Department of Obstetrics and Gynecology, Chaim Sheba Medical Center, affiliated with the Sackler Faculty of Medicine, Tel Aviv University, Tel Aviv 6997801, Israel., 5262100 Tel Hashomer, Israel

**Keywords:** Ovary, Granulosa cells, Cumulus expansion, Cumulus migration, HAS2-AS1

## Abstract

**Background:**

The cumulus expansion process is one of the LH mediated ovulatory processes. Hyaluronan synthase 2 (HAS2) regulates the synthesis of hyaluronic acid, the main component of the cumulus expansion process. Recently, the lncRNA HAS2 antisense RNA 1 (HAS2-AS1) was identified in our global transcriptome RNA-sequencing of novel ovulation associated genes. The role of HAS2-AS1 in HAS2 regulation w.as studied previously with contradictive results in different models but not in the ovary. Taken together the induction of HAS2-AS1 and the important role of HAS2 in the cumulus expansion process, we hypothesize that HAS2-AS1 regulate HAS2 expression and function in the ovary. Therefore we undertook to study the expression, regulation, and possible functional role of HAS2-AS1 in the human ovary.

**Results:**

HAS2-AS1, located within the HAS2 gene that was highly regulated in our library. We found that HAS2-AS1 express mainly in cumulus cells (CCs). Furthermore, HAS2-AS1 showed low expression in immature CCs and a significant increase expression in mature CCs. Functional studies reveal that inhibition of HAS2-AS1 by siRNA caused decrease expression of HAS2. Furthermore, inhibition of HAS2-AS1 by siRNA results in decrease migration of granulosa cells.

**Conclusions:**

Our results suggest that HAS2-AS1 is an LH/hCG target gene that plays a positive role in HAS2 expression and thus might play a role in regulating cumulus expansion and migration.

## Introduction

Critical to ovulation process is the cumulus-oocyte complex (COC) that undergos cumulus expansion. Genes that are induced following the LH surge and that are essential for proper expansion are hyaluronan synthase2 (HAS-2) and cyclooxygenase-2 (COX-2) that control the synthesis of hyaluronan and prostaglandins (such as PGE2) respectively [1, 2]. HAS2 is the main enzymes for synthesizing hyaluronic acid (HA). HA plays a critical role in vascular pathologies as it favors cell proliferation, migration, and the development of a pro-inflammatory state. In addition to hyaluronan structural backbone, there are several hyaluronan binding proteins, i.e. the proteoglycan versican [3], the serum-derived factor inter-αtrypsin inhibitor (IαI) [4] and the secreted protein tumor necrosis stimulated gene-6 (TSG-6) [5]. Therefore, the production of hyaluronan is necessary for cumulus expansion [6]. HAS2 was suggested as potential biomarkers in CCs for selection of oocytes and embryos in the IVF program [7].

Protein-coding genes account for about only 2% of the human genome, whereas the vast majority of transcripts are non-coding RNAs. Recent advances in RNA-sequencing technologies have led to the discovery of thousands of previously unannotated noncoding transcripts, including many long noncoding RNAs (lncRNAs). A growing volume of literature has proposed that lncRNAs are important factors in folliculogenesis and ovulation. However, the mechanism through which lncRNAs function remains largely unknown [8–10].

Recently, our lab applied global transcriptome RNA-sequencing approach to systematically identify novel ovulation associated genes and lncRNA [11]. One of the lncRNAs which were differentially expressed is long non-protein coding HAS2 antisense RNA 1 (HAS2-AS1), located within HAS2 gene.

HAS2-AS1 has first discovered in human and mouse osteosarcoma cells [12]. It was shown that HAS2-AS1 regulate the expression of HAS-2 in both directions. Initially, it was shown that the role of HAS2-AS1 is to suppress HAS2 mRNA levels and cell proliferation [12]. Later, HAS2-AS1 was described in the renal proximal tubular epithelial cell [13], where its expression was found in correlation with HAS2 transcription, suggesting a positive effect of HAS2-AS1 on HAS2 mRNA expression. HAS2-AS1 was also described in human aortic smooth muscle cells where it was necessary for the induction of the HAS2 gene [14].

In view of the great importance of HAS2 in the ovulation process particularly in the stages of the cumulus expansion process and the unknown role of HAS2-AS1 in HAS2 regulation in the ovary, the aim of this study was to characterize HAS2-AS1 expression and regulation in vivo and in vitro in the human ovary, to elucidate its effect on HAS2 expression in human granulosa cells and to investigate its role in the ovulatory process.

## Results

**Fig. 1:**
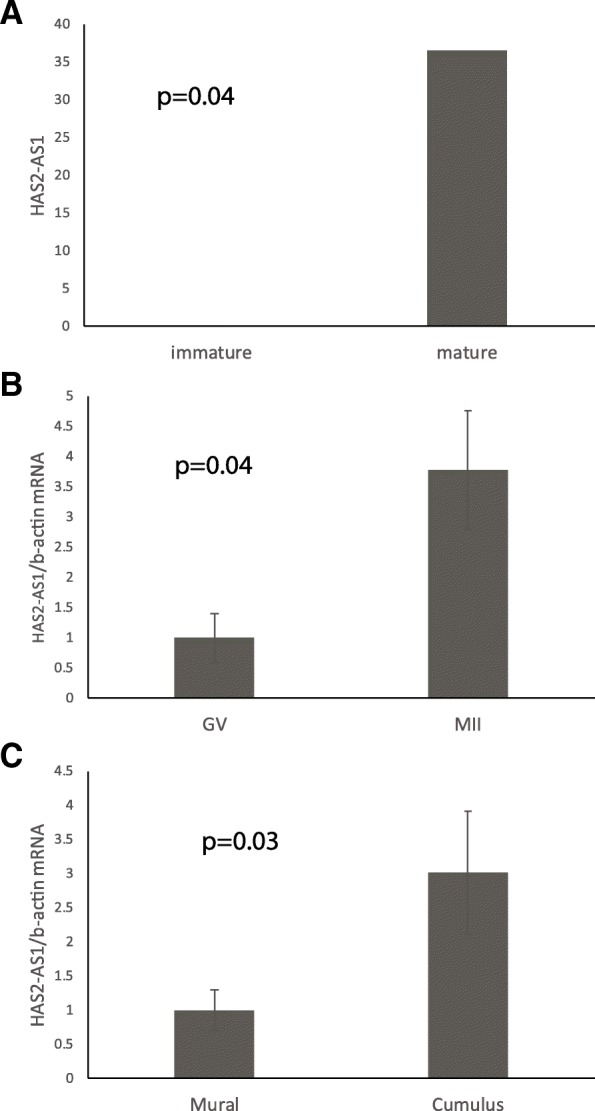
Characterization of HAS2-AS1 in human granulosa cells. (**a**) Expression of HAS2-AS1 in our library [11] of immature (compact, obtained from GV oocyte) and mature (expanded, obtained from MII oocytes) CCs. (**b**) The in vivo expression of HAS2-AS1 transcripts in CCs of expanded post-ovulatory MII COCs following IVF treatment proved 3.5-fold higher as compared with CCs of compact GV COCs following IVF treatment (*p* < 0.01). (**c**) The in vivo expression of HAS2-AS1 transcripts was also studied in granulosa cells of pre-ovulatory (> 17 mm) follicles obtained in the course of in vitro fertilization (IVF). As shown (**c**), CCs expressed higher levels of HAS2-AS1 transcripts relative to MGCs (3 fold) counterparts (*p* = 0.03). All data are normalized to b-actin expression, and represent means ± SEM of three independent experiments

As mention above, One of the lncRNA that show differential expression in our library of ovulation associated genes [11] was HAS2 long non-coding antisense 1 (HAS2-AS1). It showed low expression (below the threshold level) in immature CCs and a significant increase in expression in the mature CCs (36 reads, Fig. 1a).

To confirm the hCG dependent expression of HAS2-AS1 we compared its expression in CCs obtains from oocytes at different maturational stages. The results showed increased expression of HAS2-AS1 in CCs obtained from MII oocyte compared to CCs obtained from GV oocyte during stimulated IVF treatment (Fig. 1b).

The HAS2 role in cumulus expansion may suggest that its potential regulator, HAS2-AS1, will be expressed mainly in CCs. To characterize HAS2-AS1 expression in human ovarian follicle we compare HAS2-AS1 expression in CCs and mural granulosa cells (MGCs). The in vivo results indicate higher mRNA expression of HAS2-AS1 in CCs cells compared to MGCs (Fig. 1c).

**Fig. 2:**
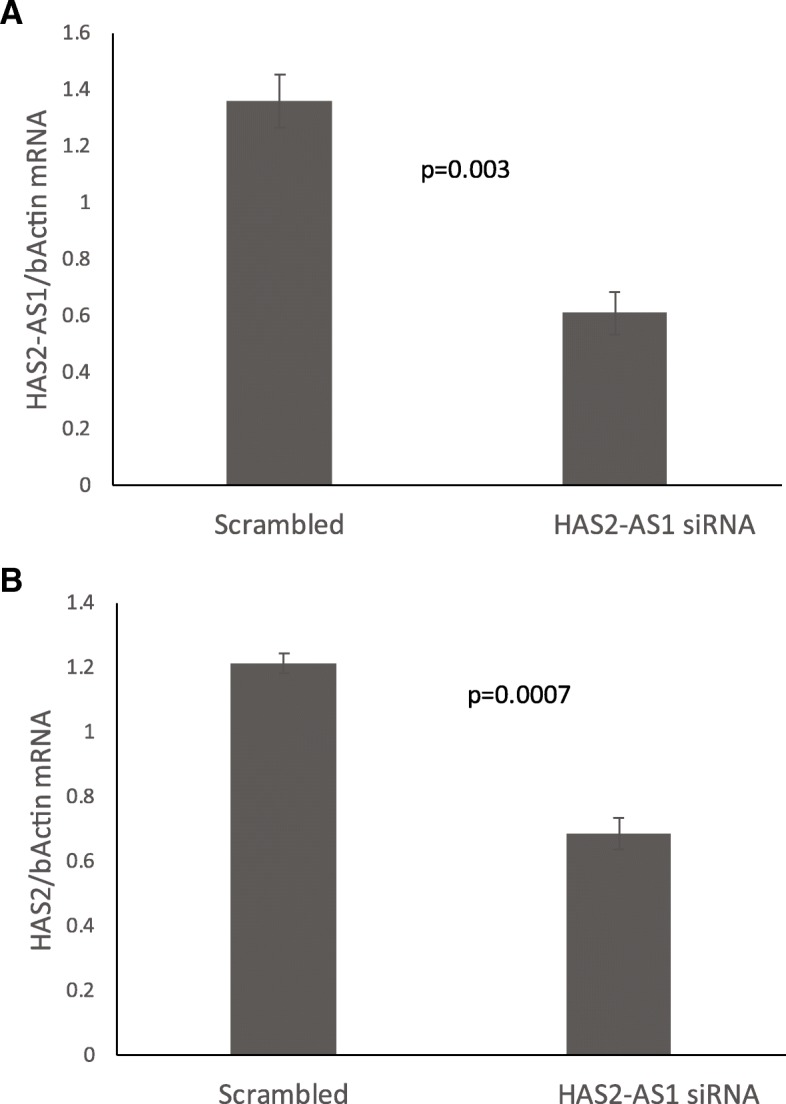
HAS2-AS1 effect on HAS2 expression. KGN cells were transfected with HAS2-AS1 siRNA or scrambled control. After 48 h cells were harvested and subjected to total RNA purification, cDNA synthesis and qRT-PCR with HAS2 and HAS2-AS1 primers. Results are means ± SEM of three independent experiments normalized to b-actin expression

The first hint for the effect of HAS2-AS1 on HAS2 expression was obtained in our library of ovulation associated genes [11] that show parallel induction of HAS2 and HAS2-AS1, suggest a positive effect. To confirm the positive effect of HAS2-AS1 on HAS2 expression, we transfected mural granulosa cell line (KGN) with HAS2-AS1 siRNA or control siRNA and 48 h later the cells were harvested. The results show that HAS2-AS1 levels (Fig. 2a) were reduced about 55% relative to control. Interestingly, HAS2 expression level (Fig. 2b) was also inhibited significantly. This result indicates and confirms that HAS2-AS1 has a positive effect on HAS2 expression.

**Fig. 3:**
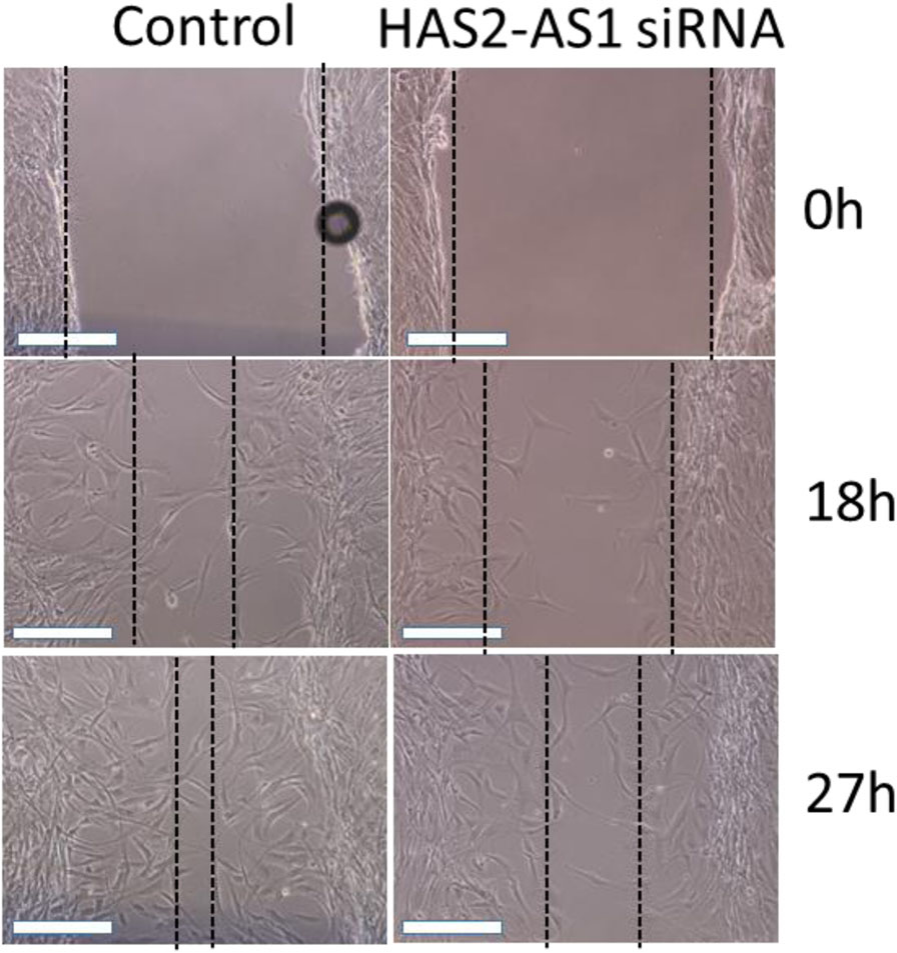
KGN cells, transfected with HAS2-AS1 siRNA or scrambled control, were grown at 95–100% confluency and starved overnight (12-14 h) in serum-free medium. During the scratch assay, FBS in the medium was set to 0.2% to inhibit apoptosis or necrosis, and at the same time, inhibit proliferation. A midline vertical scratch was performed using a 10 μl tip with a blunt surface. Images were taken 0 h, 18 h, and 27 h after incubation. Each bar represents 500 mm. The dotted lines define the areas lacking cells. Images reflect a representative experiment out of a total of at least three independent experiments

To study the role of HAS2-AS1 on HAS2 function, we examine the functional role of HAS2-AS1 in cell migration that was previously described as a HAS2 function [15]. Briefly, KGN cells, transfected with HAS2-AS1 siRNA or control siRNA, were grown to 95–100% confluency and starved overnight (12-14 h) in serum-free medium. A midline vertical scratch was performed and images were taken at 0 h and after 18 h, and 27 h of incubation. The results (Fig. 3) show that HAS2-AS1 siRNA transfected KGN cells migrate slower than the control siRNA transfected KGN cells. This result suggests a role for HAS2-AS1 in cell migration.

In summary, we showed in vivo (and ex vivo) that HAS2-AS1 is an hCG-induce gene and is positively effect HAS2 expression and function.

## Discussion

Small noncoding RNAs (ncRNAs) such as siRNAs, and miRNAs, are highly conserved and involved in transcriptional and post-transcriptional gene silencing through specific base pairing with their targets. In contrast, the lncRNAs mechanisms of gene expression regulation are numerous due to their diverse structural conformations, biochemical properties, and specific subcellular localization, resulting in both induction and suppression of their target gene [16–18].

Recently there has been a growing interest in the role of noncoding RNAs in the regulation of reproduction. Men and colleges [19] showed that StAR is a novel target of the microRNA let-7, which itself is regulated by the lncRNA H19. Overexpression of H19 stimulates StAR expression by antagonizing let-7, which inhibits StAR at the post-transcriptional level. Nakagawa [20] showed that the lncRNA Neat1 is required for corpus luteum formation and the establishment of pregnancy in mice. However, the information about the intriguing role of lncRNA in the ovulation process is still scarce and further studies are needed to learn about their role in the complex ovulatory process.

The fact that lncRNAs regulatory mechanisms are diverse may explain the opposite regulatory effects of HAS2-AS1 on HAS2 expression reported in different tissues. Our novel results show that HAS2-AS1 has a positive effect on the induction of HAS2 in human granulosa cells. The positive effect on the induction of HAS2 was shown in other tissues as well [21, 22] however, Chao et al. [23] demonstrated that HAS2-AS1 inhibits HAS2 expression in human osteosarcoma cells.

HAS2-AS1 is located on the opposite strand of the HAS2 gene, phenomena known as natural antisense (NAT) lncRNA. Although HAS2-AS1 share some sequence with HAS2, its action through direct binding is not likely because the HAS2-AS1 is expressed at much lower levels than HAS2 (data not shown). Our results showed that HAS2-AS1 positively regulates HAS2 expression. The molecular mechanism for this phenomenon might be similar to previous reports that transcription of the antisense RNA stabilizes or augments HAS2 mRNA expression via RNA/mRNA heteroduplex formation [13] and by altering the chromatin structure around the HAS2 proximal promoter [14]. It is clear that additional studies are needed to understand the regulatory mechanisms of HAS2-AS1 on HAS2 expression and hence to understand the different effect in different tissues.

In this study, we suggest for the first time that HAS2-AS expression is induced by LH/hCG. It is known that HAS2 expression is induced by LH/hCG [24] however induced expression of HAS2-AS by LH/hCG is novel. This new finding raises intriguing questions regarding how LH/hCG effects HAS2 expression and the possibility of the synergistic influence of both LH/hCG and HAS2-AS on HAS2 expression and function.

During the hyaluronan synthesis, the cumulus cells lose contact with one another and migrate outward from the oocyte along the hyaluronan polymeric backbone, a process called expansion. Although the molecular mechanisms and factors that control COC expansion have been studied extensively, much less is known about what factors control cumulus cell movement. It was shown that calpain activity was associated with cumulus cell detachment and movement by mechanisms that did not alter HAS2 expression [25]. Hyaluronic acid was shown to facilitate migration of several cells in other systems including human keratinocytes [26], human dental pulp stem cells [27], breast cancer cell lines [28], and human T-lymphocyte [29].

The role of cumulus expansion includes facilitates the extrusion of the oocyte through the ruptured follicle wall during ovulation and assists its capture by the oviductal fimbria and its entry into the oviduct. Cumulus expansion was also shown to be essential not only for ovulation but also for in vivo fertilization, and perhaps more specifically for sperm capacitation [30, 31].

We assume that inhibiting HAS2 activity using HAS2-AS1 as a target, will result in inhibition of the processes as mentioned above and therefore reducing ovulation and fertilization efficiency.

Ovulation includes several significant processes such as cumulus expansion, steroidogenesis and cytoplasmic and nuclear maturation of the oocyte. Interestingly, these processes are independent, and inhibition of one of these processes does not obligatory block the others. Accordingly, we speculated that by targeting cumulus expansion specifically, we might achieve ovulation inhibition without harming the other processes (such as steroidogenesis), and therefore less systemic side effects.

In summary, we show here for the first time that the human natural HAS-AS1 is an LH/hCG target gene that regulates HAS2 expression and function in human granulosa cells. It can be assumed that the use of this finding can lead to the development of targeted contraception with reduced systemic side effects.

## Materials and methods

### IVF protocol

Normo-ovulatory young women (< 37 years of age) undergoing IVF due to male factor infertility or genetic indication for pre-implantation genetic diagnosis were selected for this study. Subjects carrier of Fragile X disorder, endometriosis and polycystic ovary syndrome (PCOS) were excluded. Ovarian stimulation was carried out as previously described [32]. Briefly, short antagonist protocol was used, consisted of ovarian suppression with GnRH antagonists (0.25 mg/day, Cetrorelix, Cetrotide; Merck Serono International) and controlled ovarian hyperstimulation with human menopausal gonadotropin (HMG; Menopur) or recombinant FSH, either Gonal-F; Merck Serono or Puregon, MSD. The initial dose used was dependent upon age, body mass index, and previous IVF treatment history. When three or more follicles exceeded18 mm in diameter, 250 μg of hCG (Ovitrelle; Merck Serono) was administered to trigger ovulation. Transvaginal follicular aspiration was performed 36 h later with ultrasound guidance.

### Cumulus granulosa cell collection and grouping

CCs were obtained during oocyte denudation for intracytoplasmic sperm injection (ICSI) procedures. After oocyte retrieval, CCs of each oocyte were removed using hyaluronidase (SAGE) and a glass denudation pipette (Swemed). The CCs were assigned according to the related oocyte maturation status, GV, MI, or MII. The percentage of GV oocytes was 5–10%. The CCs were washed in PBS and centrifuged at 5000×g for 5 min at room temperature. The CCs from 3 to 4 subjects were pooled to generate a single replicate and the resulting pellets were stored at − 80 °C until subjected to total RNA isolation.

### Mural granulosa cell collection and grouping

MGCs were collected from aspirated follicular fluid during IVF procedures and re-suspended in a phosphate-buffered solution (PBS; Sigma-Aldrich). After allowing the cells to settle by gravity for a few minutes, the top portion of the medium was repeatedly aspirated until the medium proved clear. The cells were then centrifuged at 1000 rpm for 5 min at room temperature. Purified MGCs from large follicles (> 10 mm) of 3–4 subjects were pooled to generate a single replicate. All replicates were store at − 80 °C until subjected to total RNA purification.

### RNA extraction and qPCR

Total RNA was extracted from MGCs using a Mini/Micro RNA Isolation I kit (Zymo Research) according to the manufacturer’s instructions. RNA purity and concentration were assessed using a NanoDrop spectrophotometer (NanoDrop 2000C, Thermo Scientific). Total RNA (25 ng) from each sample was used for cDNA synthesis with a high capacity reverse transcription kit (Applied Biosystems) according to the manufacturer’s instructions in a 10 μl total volume reaction. mRNA concentrations were analyzed by real-time PCR using the StepOnePlus real-time PCR system (Applied Biosystems). The real-time PCR mix contained 1 μl of cDNA, fast SYBR Green Master Mix (Applied Biosystems), and specific primers for HAS2 or HAS2-AS1 and β-actin (housekeeping gene) in a total volume of 10 μl. Cycling parameters were: 1 cycle at 95 °C for 20 s and 40 cycles each at 95 °C for 3 s and at 60 °C for 30 s. A melting curve analysis was performed at the end of each run to ensure a single amplicon. All samples were run in duplicates. qPCR results were analyzed with StepOne software. Relative gene expression was calculated using the delta-delta Ct method. Details of the primers are shown in Table 1.

**Table 1:** Primers sequences

Organism	Gene	Primer sequence	Accession number
*Homo sapiens*	β-Actin (ACTB)	sense, 5′ - TTGCCGACAGGATGCAGAA	NM_001101.3
		antisense, 5′ - GCTCAGGAGGAGCAATGATCTT	
	HAS2	sense, 5′ – AGCCTTCAGAGCACTGGGACGA	NM_005328.3
		antisense, 5′ - ACAGATGAGGCTGGGTCAAGCA	
	HAS2-AS1	sense, 5′ – AGGGGTGGACTTCTTTGGAAC	NR_002835.2
		antisense, 5′ - CCAAACAGCTCCTTGTGCG	

### KGN siRNA

KGN cells [33] were transfected, using Dharmafect1 (Dharmacon Inc.), with HAS2-AS1 siRNA (SMARTpool: Lincode Human HAS2-AS1 siRNA; NR_002835; R-187921-00-0005) or scrambled control (Dharmacon Lincode Non-targeting siRNA #1) according to the manufacture instruction. After 48 h cells were harvested and subjected to total RNA purification, cDNA and qRT-PCR with HAS2 and HAS2-AS1 primers.

### In vitro scratch assay

KGN cells [33] were transfected, using Dharmafect1 (Dharmacon Inc.), with HAS2-AS1 siRNA (SMARTpool: Lincode Human HAS2-AS1 siRNA; NR_002835; R-187921-00-0005) or scrambled control (Dharmacon Lincode Non-targeting siRNA #1) according to the manufacture instruction. After 48 h cells were grown at 95–100% confluency, and starve overnight (12-14 h) in serum-free medium. During scratching assay, FBS in the medium was set to 0.2% to inhibit apoptosis and necrosis, and at the same time, inhibit proliferation. A midline vertical scratch was performed using a 10 μl tip with a blunt surface. Pictures were taken immediately (oh), after 18 h, and 27 h of incubation.

## References

[1] Hizaki H, Segi E, Sugimoto Y, Hirose M, Saji T, Ushikubi F (1999). Abortive expansion of the cumulus and impaired fertility in mice lacking the prostaglandin E receptor subtype EP(2). Proc Natl Acad Sci U S A.

[2] Richards JS, Pangas SA (2010). The ovary: basic biology and clinical implications. J Clin Invest.

[3] Russell DL, Robker RL (2007). Molecular mechanisms of ovulation: co-ordination through the cumulus complex. Hum Reprod Update.

[4] Hess KA, Chen L, Larsen WJ (1999). Inter-alpha-inhibitor binding to hyaluronan in the cumulus extracellular matrix is required for optimal ovulation and development of mouse oocytes. Biol Reprod.

[5] Fulop C, Szanto S, Mukhopadhyay D, Bardos T, Kamath RV, Rugg MS (2003). Impaired cumulus mucification and female sterility in tumor necrosis factor-induced protein-6 deficient mice. Development..

[6] Richards JS (2005). Ovulation: new factors that prepare the oocyte for fertilization. Mol Cell Endocrinol.

[7] Shepel EA, Voznesenskaya TY, Blashkiv TV, Yanchii RI (2016). CUMULUS CELL GENES AS POTENTIAL BIOMARKERS OF OOCYTE AND EMBRYO DEVELOPMENTAL COMPETENCE. Fiziolohichnyi zhurnal (Kiev, Ukraine : 1994).

[8] Kurokawa R (2011). Long noncoding RNA as a regulator for transcription. Prog Mol Subcell Biol.

[9] Yan B, Wang Z (2012). Long noncoding RNA: its physiological and pathological roles. DNA Cell Biol.

[10] Kim ED, Sung S (2012). Long noncoding RNA: unveiling hidden layer of gene regulatory networks. Trends Plant Sci.

[11] Yerushalmi GM, Salmon-Divon M, Yung Y, Maman E, Kedem A, Ophir L (2014). Characterization of the human cumulus cell transcriptome during final follicular maturation and ovulation. Mol Hum Reprod.

[12] Chao H, Spicer AP (2005). Natural antisense mRNAs to hyaluronan synthase 2 inhibit hyaluronan biosynthesis and cell proliferation. J Biol Chem.

[13] Michael DR, Phillips AO, Krupa A, Martin J, Redman JE, Altaher A (2011). The human hyaluronan synthase 2 (HAS2) gene and its natural antisense RNA exhibit coordinated expression in the renal proximal tubular epithelial cell. J Biol Chem.

[14] Vigetti D, Deleonibus S, Moretto P, Bowen T, Fischer JW, Grandoch M (2014). Natural antisense transcript for hyaluronan synthase 2 (HAS2-AS1) induces transcription of HAS2 via protein O-GlcNAcylation. J Biol Chem.

[15] Akison LK, Alvino ER, Dunning KR, Robker RL, Russell DL (2012). Transient invasive migration in mouse cumulus oocyte complexes induced at ovulation by luteinizing hormone. Biol Reprod.

[16] Jandura A, Krause HM. The New RNA World: Growing Evidence for Long Noncoding RNA Functionality. Trends Genet. 2017;33(10). Epub 2017 Sep 1. 10.1016/j.tig.2017.08.002 28870653

[17] Liu SJ, et al. Science. 2016. 10.1126/science.aah7111. PMID:27980086.

[18] Long Y, Wang X, Youmans DT, Cech TR. How do lncRNAs regulate transcription? Sci Adv. 2017;3(9) Epub 2017 Sep 27. 10.1126/sciadv.aao2110 PMC5617379 28959731

[19] Men Y, Fan Y, Shen Y, Lu L, Kallen AN (2017). The steroidogenic acute regulatory protein (StAR) is regulated by the H19/let-7 Axis. Endocrinology..

[20] Nakagawa S, Shimada M, Yanaka K, Mito M, Arai T, Takahashi E (2014). The lncRNA Neat1 is required for corpus luteum formation and the establishment of pregnancy in a subpopulation of mice. Development..

[21] Kedem A, Yung Y, Yerushalmi GM, Haas J, Maman E, Hanochi M (2014). Anti Mullerian hormone (AMH) level and expression in mural and cumulus cells in relation to age. J Ovarian Res.

[22] Yung Y, Aviel-Ronen S, Maman E, Rubinstein N, Avivi C, Orvieto R (2014). Localization of luteinizing hormone receptor protein in the human ovary. Mol Hum Reprod.

[23] Haas J, Ophir L, Barzilay E, Machtinger R, Yung Y, Orvieto R (2016). Standard human chorionic gonadotropin versus double trigger for final oocyte maturation results in different granulosa cells gene expressions: a pilot study. Fertil Steril.

[24] Stock AE, Bouchard N, Brown K, Spicer AP, Underhill CB, Dore M (2002). Induction of hyaluronan synthase 2 by human chorionic gonadotropin in mural granulosa cells of equine preovulatory follicles. Endocrinology..

[25] Kawashima I, Liu Z, Mullany LK, Mihara T, Richards JS, Shimada M (2012). EGF-like factors induce expansion of the cumulus cell-oocyte complexes by activating calpain-mediated cell movement. Endocrinology..

[26] Shi H, Asher C, Yung Y, Kligman L, Reuveny E, Seger R (2002). Casein kinase 2 specifically binds to and phosphorylates the carboxy termini of ENaC subunits. Eur J Biochem.

[27] Shi H, Asher C, Chigaev A, Yung Y, Reuveny E, Seger R (2002). Interactions of beta and gamma ENaC with Nedd4 can be facilitated by an ERK-mediated phosphorylation. J Biol Chem.

[28] Yao Z, Flash I, Raviv Z, Yung Y, Asscher Y, Pleban S (2001). Non-regulated and stimulated mechanisms cooperate in the nuclear accumulation of MEK1. Oncogene..

[29] Yung Y, Yao Z, Aebersold DM, Hanoch T, Seger R (2001). Altered regulation of ERK1b by MEK1 and PTP-SL and modified Elk1 phosphorylation by ERK1b are caused by abrogation of the regulatory C-terminal sequence of ERKs. J Biol Chem.

[30] Van Soom A, Tanghe S, De Pauw I, Maes D, de Kruif A (2002). Function of the cumulus oophorus before and during mammalian fertilization. Reprod Domest Anim.

[31] Yokoo M, Sato E. Cumulus-oocyte complex interactions during oocyte maturation. Int Rev Cytol 2004;235:251–291. PubMed PMID: 15219785. Epub 2004/06/29. eng. 10.1016/S0074-7696(04)35006-0 15219785

[32] Hourvitz A, Lerner-Geva L, Elizur SE, Baum M, Levron J, David B (2006). Role of embryo quality in predicting early pregnancy loss following assisted reproductive technology. Reprod BioMed Online.

[33] Nishi Y, Yanase T, Mu Y, Oba K, Ichino I, Saito M (2001). Establishment and characterization of a steroidogenic human granulosa-like tumor cell line, KGN, that expresses functional follicle-stimulating hormone receptor. Endocrinology..

